# The value of quality feedback in formative OSCEs

**DOI:** 10.1080/10872981.2017.1353877

**Published:** 2017-07-18

**Authors:** Adnan Ahmed, Mohammad Alwaheedy, Ahamodur Choudhury, Sheikh Naheed Chowdhury, Maaz Khan, Basir Kunduzi, Abdul Rahyead

**Affiliations:** ^a^ King’s College London, Guys Kings St Thomas’ School of Medicine, London, UK

Dear Sir,

We would firstly like to credit Bernard et al. [1] for their interesting research into such a topical aspect of medical education currently, one which we can relate to personally as students who share an interest in medical education. The role of feedback in promoting student reflection and subsequent improvement in the setting of formative objective structured clinical examinations (OSCEs) is emphasised well in this paper and we applaud the authors for describing a variety of feedback systems.

We found it interesting that the current study reported that only 64% of students reviewed their feedback when left to their own accord, and also surprising that the frequency of feedback review was not significantly associated with improved performance in summative OSCEs. Although the authors raise valid points to explain these findings, we believe that there may be more fundamental issues with the feedback content itself. As senior medical students engaged in improving medical education we are firm believers that the quality of feedback is as important as the mode of delivery in determining feedback uptake. Indeed, previous reports suggest that feedback given can be of low quality in terms of being difficult for students to decipher and lacking steps to facilitate implementation of the feedback into practice [2].

We believe that for feedback to be of high quality, two conditions need to be met: firstly, the feedback content itself should be presented in a way that facilitates actionable change and, secondly, the feedback should be delivered in an easily accessible manner which encourages revision by students. We propose that the quality of feedback is a key factor the authors of this paper have yet to explore in detail.

We feel that this first condition is an important point to focus on, as there is often a difference in expectations between students and examiners [3], which may be further exacerbated by the inherently artificial nature of the OSCE setting. We believe that making students better aware of the specific expectations of the examiner by providing steps to attain an ‘ideal’ performance, without revealing the specific content of the examination itself, will help to combat any such discrepancies and to promote student confidence. With better awareness of these expectations, students can, during self-review, focus on improving their particular deficiencies according to the curriculum, which can both encourage feedback uptake and improve clinical practice.

Although several modes of feedback delivery were explored in the current study, we feel the structure of feedback given may be worth considering further. Lunsford’s analysis of feedback delivery methods suggests high quality feedback should consist of an optimum of three well-thought, detailed comments that cover not only strengths and weaknesses of a student’s performance but also the aforementioned actionable advice, all written in a non-authoritative style [4]. We feel this style of feedback would equip students well to target areas for improvement.

We are aware that large institutional cohort sizes pose a significant challenge to the implementation of the type of feedback model described here. Various approaches can be used to combat this. For example, small group breakout sessions may be organised, or classroom technologies used to collate student responses in order to stimulate discussion regarding common concerns. This would allow exploration of common student doubts without straining resources.

We believe that feedback to students should not be viewed as transfer of facts, but rather a dialogue that allows students to discern the gaps in their knowledge, develop an understanding of competency requirements, and form a plan of action moving forward. It is important to acknowledge that high quality feedback underpins any such dialogue and that much of this responsibility falls upon the examiner. This raises the issue of ensuring adequate examiner training and standardisation, which would need to be addressed in further studies [5]. We write this letter because we are firm believers that students should be given the maximum opportunity to develop their skills, and we advocate a greater push towards quality-oriented feedback models to better achieve this following OSCEs.
